# Supplementary material for: Tertiary lymphoid structures and B cells determine clinically relevant T cell phenotypes in ovarian cancer
Source: Nat Commun. 2024 Mar 21;15:2528. doi: 10.1038/s41467-024-46873-w (PMC10957872; doi:10.1038/s41467-024-46873-w)
Supplement: Supplementary file 1 — Supplementary Information [file 41467_2024_46873_MOESM1_ESM.pdf]

Supplementary Figures

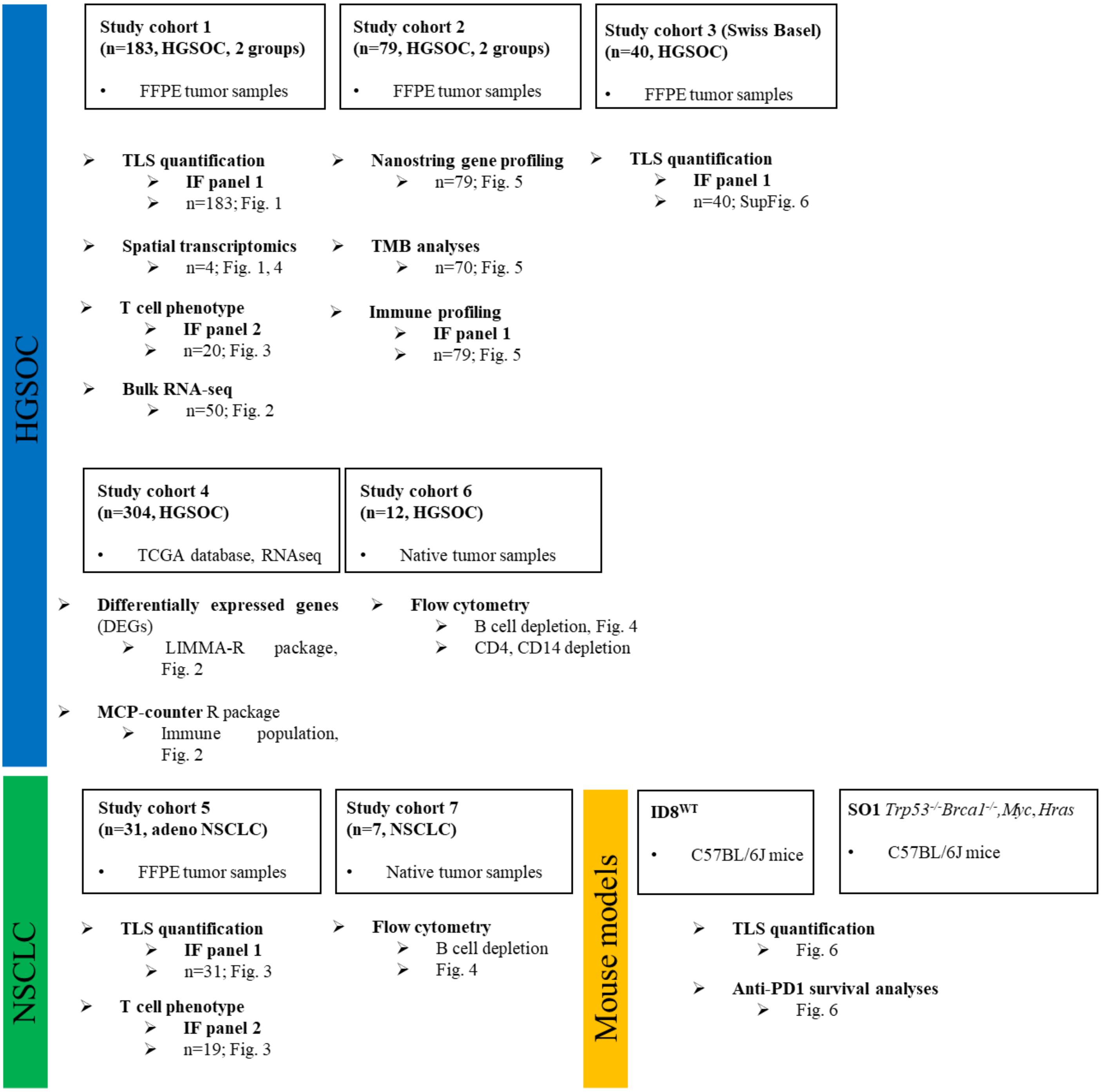

Supplementary Figure 1. **Experimental design**

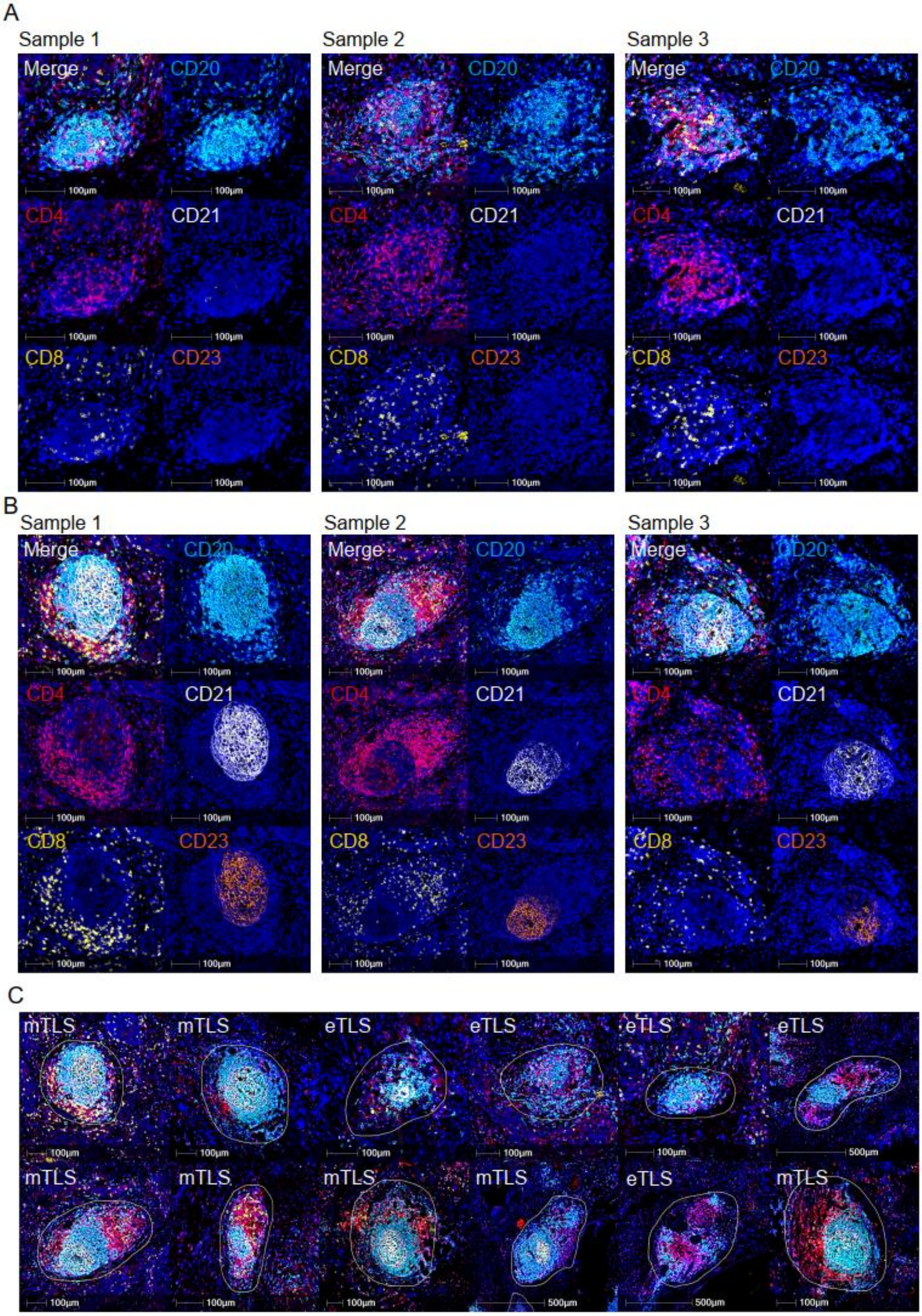

Supplementary Figure 2. **Spatial distribution of eTLS and mTLS. (A, B)** Representative images of eTLS **(A)** and mTLS **(B)** using immunofluorescence of CD4, CD8, CD20, CD21, CD23, DC-LAMP and GZMB staining in 6 HGSOC patients. **(C)** Representative image of TLS areas quantification in HALO software in 6 HGSOC patients. Scale bar 100 and 500 µm.

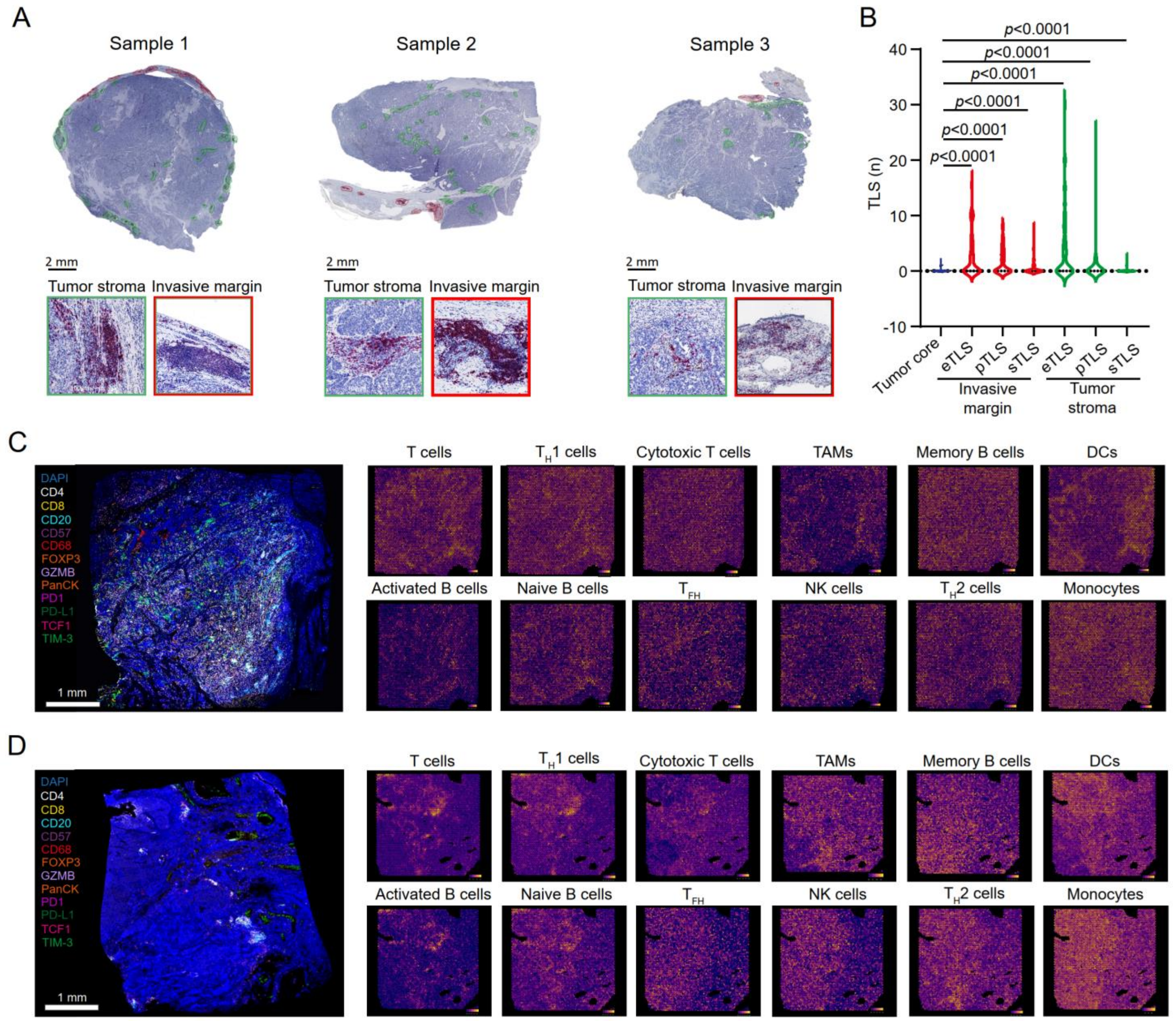

Supplementary Figure 3. **The localization and spatial immune composition of tertiary lymphoid structures (TLS) in HGSOC. (A, B)** Representative images of 3 HGSOC samples **(A)** and distribution violin plot **(B)** of early TLS (eTLS; $CD4^+CD8^+CD20^+CD21^-CD23^-$), TLS with primary follicle (pTLS; $CD4^+CD8^+CD20^+CD21^+CD23^-$), and secondary TLS (sTLS; $CD4^+CD8^+CD20^+CD21^+CD23^+$) within invasive margin, tumor core and stroma of 115 HGSOC determined by immunostaining. Scale bar 2 mm. Statistical significance was calculated by two-sided Mann-Whitney test. p values are indicated. **(C, D)** Spatial co-localization of TLS with gene signatures of T cells, T_H1 cells, cytotoxic T cells, activated and naïve B cells, T follicular cells (T_{FH}) cells and tumor associated macrophages (TAMs), memory B cells, dendritic cells (DCs), natural killer (NK) cells, T_H2 cell and monocytes in 2 tumor samples from study group 1, determined by Visium transcriptomic and "metagene" markers. Immunofluorescence staining for CD68, CD8, PD-L1,

FoxP3, TCF1, CD57, PanCK, PD1, CD4, CD20, GZMB and TIM3 staining (*immunofluorescence panel 2*) on FFPE TLS^+ tumor used for the spatial transcriptomic assay delineate pathologically identified TLS areas.

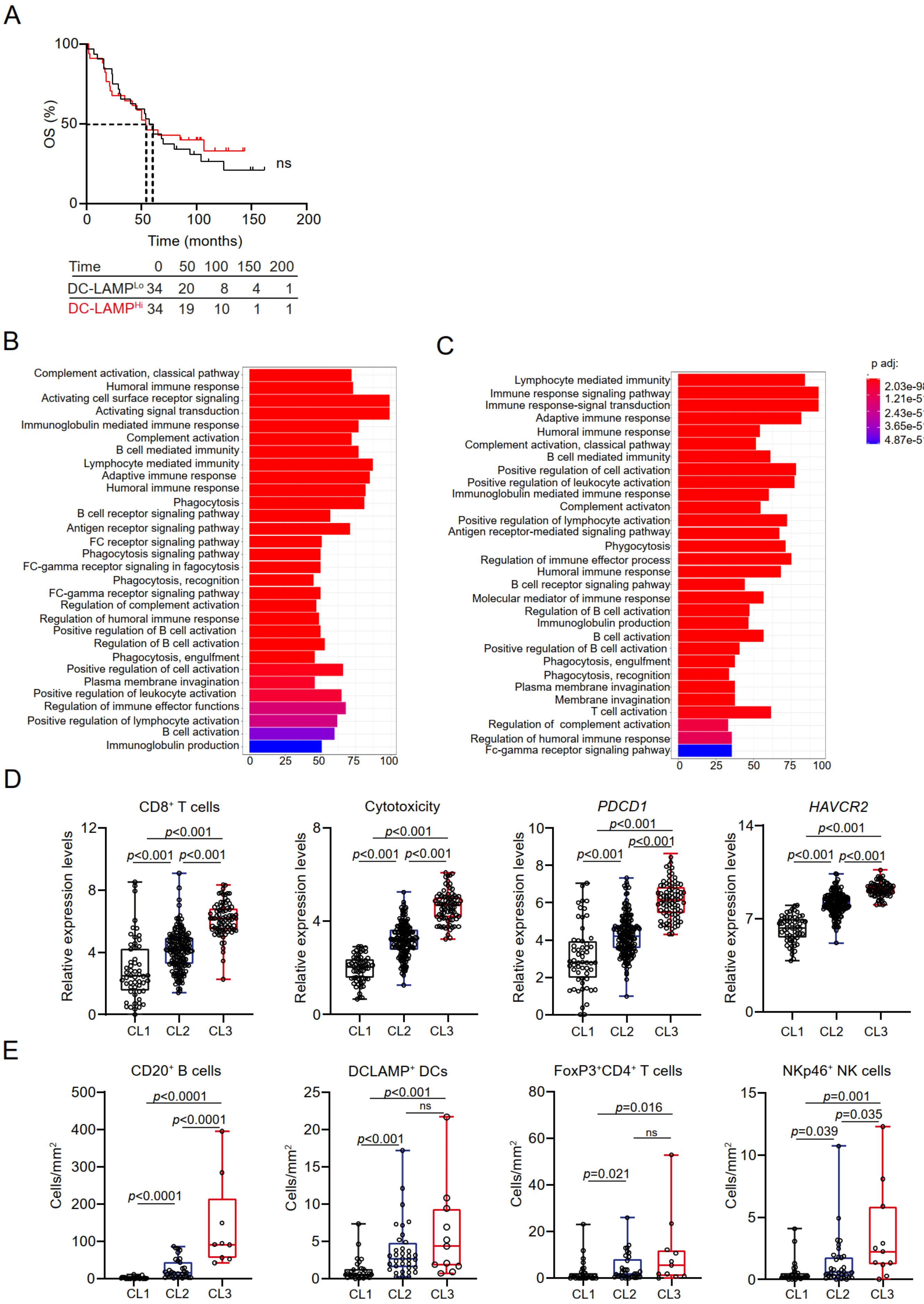
A
OS (%)
Time (months)
ns
Time 0 50 100 150 200
DC-LAMPLo 34 20 8 4 1
DC-LAMPHi 34 19 10 1 1
B
Complement activation, classical pathway
Humoral immune response
Activating cell surface receptor signaling
Activating signal transduction
Immunoglobulin mediated immune response
Complement activation
B cell mediated immunity
Lymphocyte mediated immunity
Adaptive immune response
Humoral immune response
Phagocytosis
B cell receptor signaling pathway
Antigen receptor signaling pathway
FC receptor signaling pathway
Phagocytosis signaling pathway
FC-gamma receptor signaling in fagocytosis
Phagocytosis, recognition
FC-gamma receptor signaling pathway
Regulation of complement activation
Regulation of humoral immune response
Positive regulation of B cell activation
Regulation of B cell activation
Phagocytosis, engulfment
Positive regulation of cell activation
Plasma membrane invagination
Positive regulation of leukocyte activation
Regulation of immune effector functions
Positive regulation of lymphocyte activation
B cell activation
Immunoglobulin production
C
p adj:
2.03e-98
1.21e-51
2.43e-51
3.65e-51
4.87e-51
Lymphocyte mediated immunity
Immune response signaling pathway
Immune response-signal transduction
Adaptive immune response
Humoral immune response
Complement activation, classical pathway
B cell mediated immunity
Positive regulation of cell activation
Positive regulation of leukocyte activation
Immunoglobulin mediated immune response
Complement activaton
Positive regulation of lymphocyte activation
Antigen receptor-mediated signaling pathway
Phygocytosis
Regulation of immune effector process
Humoral immune response
B cell receptor signaling pahway
Molecular mediator of immune response
Regulation of B cell activation
Immunoglobulin production
B cell activation
Positive regulation of B cell activation
Phagocytosis, engulfment
Phagocytosis, recognition
Plasma membrane invagination
Membrane invagination
T cell activation
Regulation of complement activation
Regulation of humoral immune response
Fc-gamma receptor signaling pahway
D
CD8+ T cells
Cytotoxicity
PDCD1
HAVCR2
Relative expression levels
p<0.001
CL1 CL2 CL3
E
CD20+ B cells
DCLAMP+ DCs
FoxP3+CD4+ T cells
NKp46+ NK cells
Cells/mm2
p<0.0001
p<0.001
ns
p=0.016
p=0.021
p=0.001
p=0.035
p=0.039
CL1 CL2 CL3

Supplementary Figure 4. **The clinical impact of tertiary lymphoid structures (TLSs) on development of antitumor immunity in HGSOC. (A)** Overall survival (OS) of 68 patients (Study cohort 1) based on median stratification of density of DC-LAMP$^+$ dendritic cells in non-TLS (nTLS) areas of tumor samples. Survival curves were estimated by the Kaplan-Meier method, and differences between groups were evaluated using log-rank test. Number of patients at risk and *p* values are reported. **(B, C)** Gene enrichment studies based on the enrichGo function from ClusterProfiler showing gene signatures overrepresented in tumor samples of patients from Cluster 2 with only early TLS (eTLS) **(C)** and Cluster 3 with both eTLS and mature TLS (mTLS) development **(D)** compared to tumor samples without TLS development (Cluster 1), determined by RNA sequencing in 53 HGSOC samples (see Fig.2A; Study cohort 1). TLS density was determined by immunofluorescence staining **(D)** Gene expression signature associated with CD8$^+$ T cells, cytotoxicity, and relative gene expression levels of *PDCD1* and *HAVCR2* across 304 HGSOC patients from TCGA public database (Study cohort 4) separated into 3 clusters (CL1: n=142; CL2: n=81; CL3: n=81) based on unsupervised hierarchical clustering from Figure 2C. Box plots: lower quartile, median, upper quartile; whiskers, minimum, maximum. Statistical significance was calculated by two-sided Mann-Whitney test. *p* values are indicated. ns, not significant. **(E)** Density of CD20$^+$ B cells, DC-LAMP$^+$ DCs, CD4$^+$FoxP3$^+$ cells and NKp46$^+$ NK cells in tumor samples of HGSOC patients separated into 3 clusters (Study cohort 1; CL1, no TLS development, n=24; CL2, only eTLS development, n=32; CL3, both eTLS and mTLS development, n=9), as determined by immunostaining. Box plots: lower quartile, median, upper quartile; whiskers, minimum, maximum. Statistical significance was calculated by two-sided the Mann–Whitney test. *p* values are indicated.

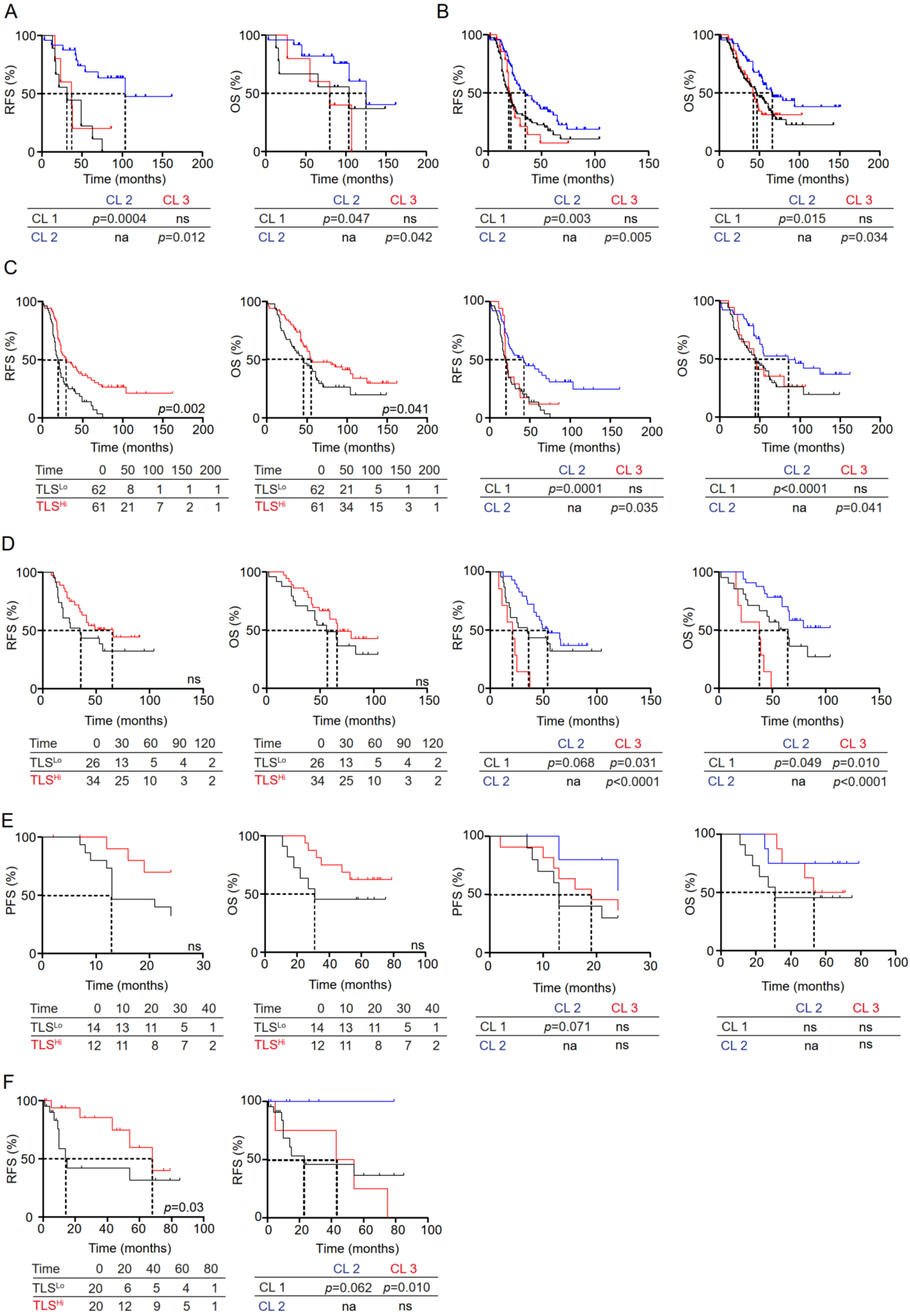
A
RFS (%)
OS (%)
Time (months)
CL 2 CL 3
CL 1 p=0.0004 ns
CL 2 na p=0.012
CL 2 CL 3
CL 1 p=0.047 ns
CL 2 na p=0.042
B
RFS (%)
OS (%)
Time (months)
CL 2 CL 3
CL 1 p=0.003 ns
CL 2 na p=0.005
CL 2 CL 3
CL 1 p=0.015 ns
CL 2 na p=0.034
C
RFS (%)
p=0.002
OS (%)
p=0.041
Time (months)
Time 0 50 100 150 200
TLSLo 62 8 1 1 1
TLSHi 61 21 7 2 1
Time 0 50 100 150 200
TLSLo 62 21 5 1 1
TLSHi 61 34 15 3 1
CL 2 CL 3
CL 1 p=0.0001 ns
CL 2 na p=0.035
CL 2 CL 3
CL 1 p<0.0001 ns
CL 2 na p=0.041
D
RFS (%)
OS (%)
ns
Time (months)
Time 0 30 60 90 120
TLSLo 26 13 5 4 2
TLSHi 34 25 10 3 2
Time 0 30 60 90 120
TLSLo 26 13 5 4 2
TLSHi 34 25 10 3 2
CL 2 CL 3
CL 1 p=0.068 p=0.031
CL 2 na p<0.0001
CL 2 CL 3
CL 1 p=0.049 p=0.010
CL 2 na p<0.0001
E
PFS (%)
OS (%)
ns
Time (months)
Time 0 10 20 30 40
TLSLo 14 13 11 5 1
TLSHi 12 11 8 7 2
Time 0 10 20 30 40
TLSLo 14 13 11 5 1
TLSHi 12 11 8 7 2
CL 2 CL 3
CL 1 p=0.071 ns
CL 2 na ns
CL 2 CL 3
CL 1 ns ns
CL 2 na ns
F
RFS (%)
p=0.03
Time (months)
Time 0 20 40 60 80
TLSLo 20 6 5 4 1
TLSHi 20 12 9 5 1
CL 2 CL 3
CL 1 p=0.062 p=0.010
CL 2 na ns

Supplementary Figure 5. **The clinical relevance of TLS formation and maturation in 4 independent cohorts of HGSOC patients. (A, B)** Relapse-free survival (RFS) and overall survival (OS) of HGSOC patients based on stratification into 3 clusters with no TLS development (CL1, n=9), only eTLS development (CL2, n=24) and both eTLS and mTLS development (CL3, n=5) in early (stage I+II; n=38) **(A)** and late (stage III+IV; n=171, CL1: n=74; CL2: n=66; CL3: n=29) **(B)** HGSOC patients (Study cohort 1 and 2; see Suppl. Table 1). **(C-F)** RFS and OS of HGSOC patients based on median stratification of total TLS and based on stratification into 3 clusters with no TLS development (CL1), only eTLS development (CL2) and both eTLS and mTLS development (CL3) from individual cohorts and groups, presented independently: **(C)** 123 HGSOC patients (Study cohort 1, group 1; Suppl. Table 1; CL1: n=52; CL2: n=52; CL3: n=17). **(D)** 60 HGSOC patients (Study cohort 1, group 2; Suppl. Table 1; CL1: n=21; CL2: n=32; CL3: n=7). **(E)** 26 HGSOC patients (Study cohort 2, group 3; Suppl. Table 2; CL1: n=11; CL2: n=10; CL3: n=6). **(F)** 40 HGSOC patients (Study cohort 3; Suppl. Table 5; CL1: n=23; CL2: n=8; CL3: n=4). Survival curves were estimated by the Kaplan-Meier method, and differences between groups were evaluated using log-rank test. Number of patients at risk and *p* values are reported.

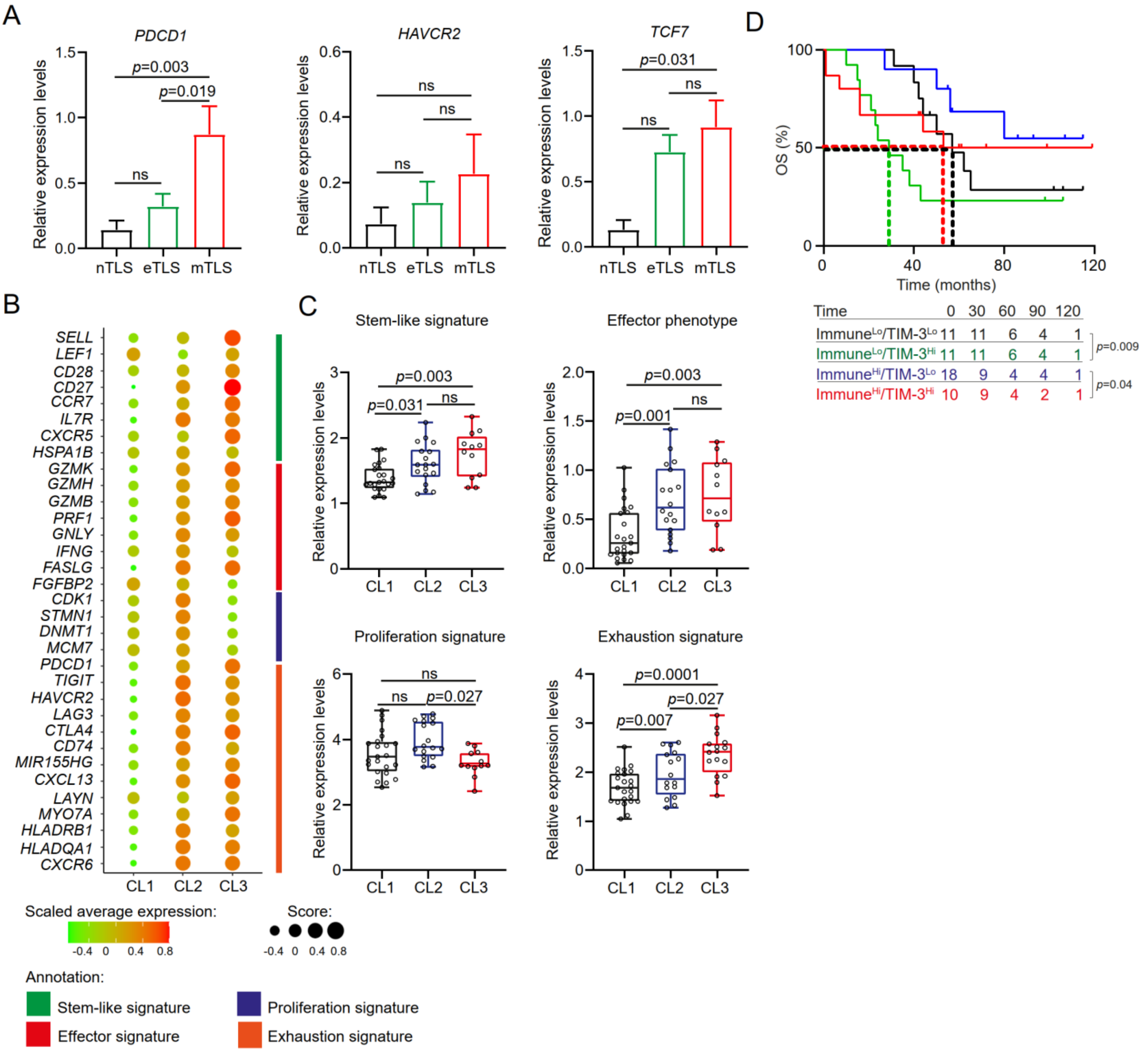

Supplementary Figure 6. **TLS formation and maturation impact the T cell phenotype in HGSOC. (A)** Relative expression levels of *PDCD1*, *HAVCR2*, *TCF7* outside TLS structures (nTLS), within early TLS (eTLS) areas and mature TLS (mTLS) areas as determined by Visium Transcriptomic in 3 HGSOC tumor samples (Study cohort 1). Statistical significance was calculated by two-sided Mann-Whitney test. *p* values are indicated. ns, not significant. **(B, C)** Relative expression bubble plot **(B)** and box plots **(C)** of selected genes or respective gene signatures known to be associated with different stages of T cell differentiation in 53 HGSOC patients (Study cohort 1; CL1: n=23; CL2: n=18; CL3: n=12). The colour of each dot represents the average normalized expression from high (red) to low (green). The size of ach dot represents the percentage of positive cells for each gene. Box plots: lower quartile, median, upper quartile; whiskers, minimum,

maximum. Statistical significance was calculated by two-sided Mann-Whitney test. *p* values are indicated. ns, not significant. **(D)** Overall survival (OS) of HGSOC patients based on median density of $TIM3^+PD1^+CD8^+$ cells and immune infiltrate. Survival curves were estimated by the Kaplan-Meier method, and differences between groups were evaluated using log-rank test. Number of patients at risk and *p* values are reported.

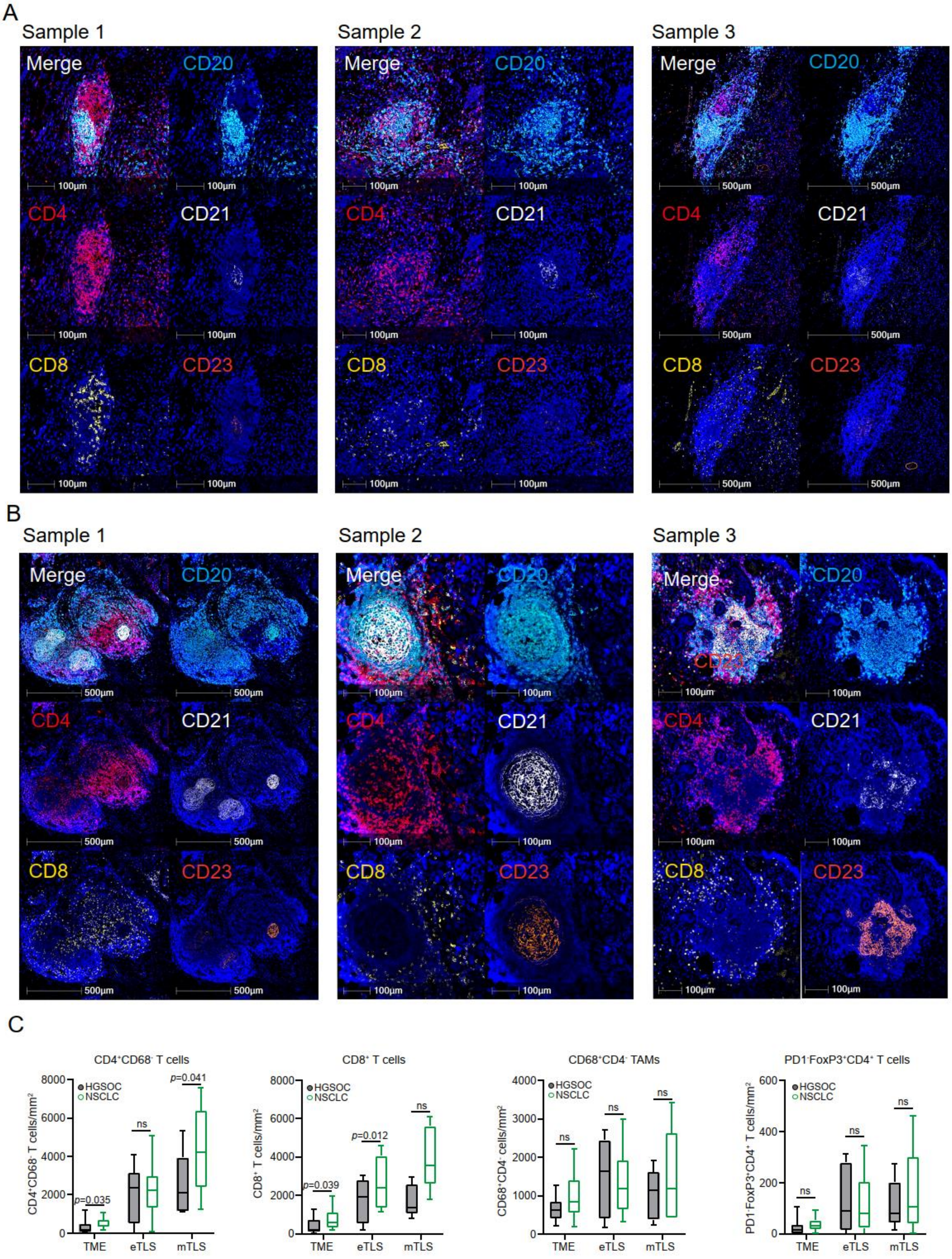

Supplementary Figure 7. **TLS formation and maturation in HGSOC and NSCLC patients.** Representative images of mature TLS (mTLS) in HGSOC (n=3) **(A)** and NSCLC (n=3) **(B)** using immunofluorescence of CD4, CD8, CD20, CD21, CD23, DC-LAMP and GZMB staining. Scale bar 100 and

500 µm. **(C)** Density of CD4$^+$CD68$^-$T cells, CD8$^+$ T cells, CD68$^+$CD4$^-$ tumor associated macrophages and PD1$^-$FoxP3$^+$CD4$^+$ T cells within complete tumor microenvironment (TME, including TLS), eTLS and mTLS of HGSOC (Study cohort 1; n=17) and NSCLC samples (Study cohort 5; n=10) as determined by immunofluorescence. Box plots: lower quartile, median, upper quartile; whiskers, minimum, maximum. Statistical significance was calculated by two-sided Mann–Whitney test. *p* values are indicated.

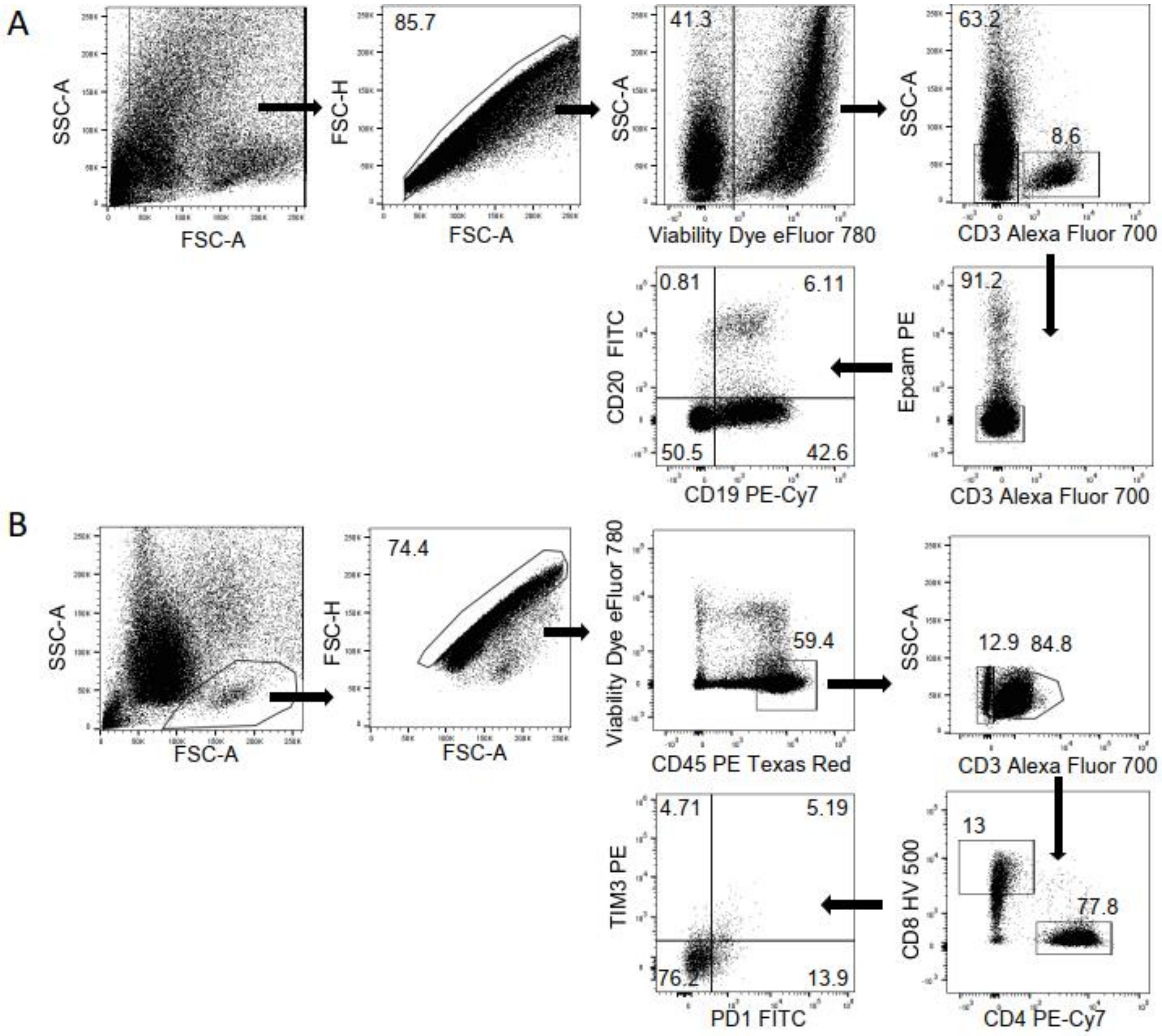

Supplementary Figure 8. **Gating strategy. (A)** Detection of relative numbers of CD19$^+$CD20$^+$ B cells and **(B)** relative numbers of PD1$^+$TIM3$^-$CD8$^+$ and PD1$^+$TIM3$^+$CD8$^+$ T cells.

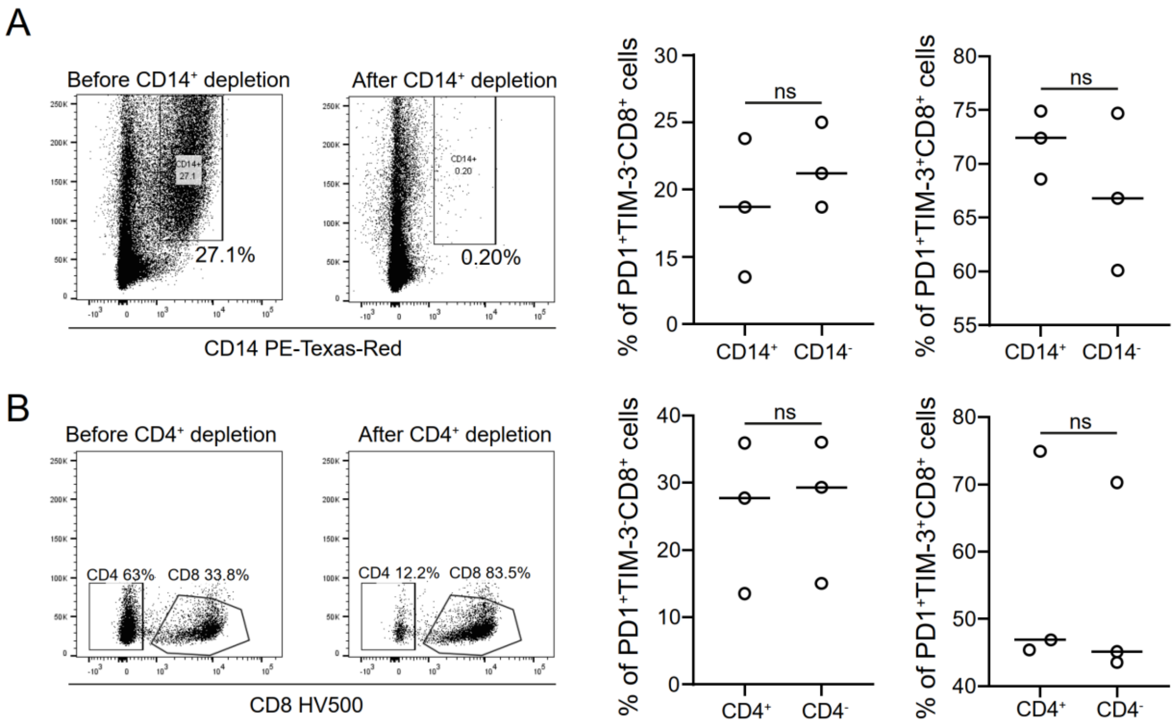

Supplementary Figure 9. **The impact of intratumoral CD4$^+$ and CD14$^+$ on CD8$^+$ T cells phenotype in HGSOC. (A, B)** Representative dot plot and flow cytometry analyses for frequency of TIM3$^+$PD1$^+$ and TIM3$^-$PD1$^+$CD8$^+$ T cells before and after CD4$^+$ cells depletion **(A)** and CD14$^+$ cells depletion **(B)** from native HGSOC tumor tissue (n=5, Study cohort 6). Statistical significance was calculated by two-sided Wilcoxon matched pairs signed rank test. *p* values are indicated.

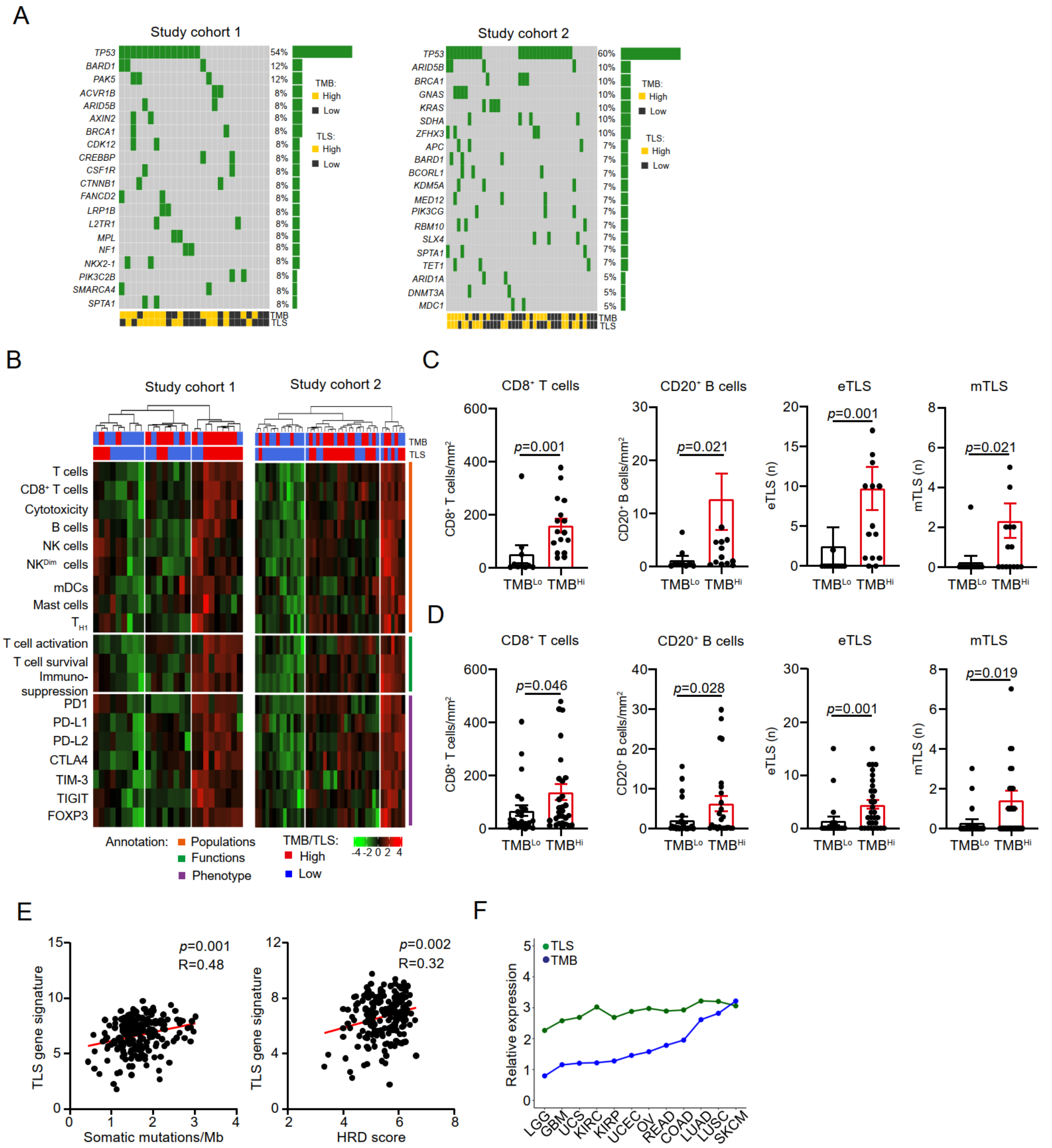

Supplementary Figure 10. **TMB positively correlates with formation of mature TLS structures in HGSOC. (A)** Oncoplot showing the profile of somatic mutations in 2 independent cohorts of 26 and 53 HGSOC patients' samples, further annotated by TMB determined by TrueSighOnco500 and TLS numbers as determined by immunofluorescence staining (Study cohort 2). **(B)** Unsupervised hierarchical clustering of gene signatures related to immune populations (orange), immune functions (green) and immune phenotype (purple), as determined by PanCancer Immune Profiling from Nanostring and further annotated by TLS and TMB in 2 independent groups of 26 (TMB^{Lo}: n=10, TMB^{Hi}: n=16) and 53 (TMB^{Lo}: n=25, TMB^{Hi}: n=28)

HGSOC patients (Study cohort 2). **(C, D)** Density of $CD8^+$ T cells, $CD20^+$ B cells, early TLS (eTLS) and mature TLS (mTLS) numbers in TMB low (TMB^{Lo}) and high (TMB^{Hi}) tumors from 2 independent groups of HGSOC patients (Study cohort 2). Box plots: lower quartile, median, upper quartile; whiskers, minimum, maximum. Statistical significance was calculated by two-sided Mann-Whitney test. *p* values are indicated. **(E)** Correlation between the TLS gene signature (*CCL2, CCL3, CCL4, CCL5, CCL8. CCL18, CCL19, CCL21, CXCL9, CXCL10, CXCL11, CXCL13*) and somatic mutations and HRD score in 304 HGSOC patients from the TCGA public database. R, Pearson correlation coefficient. **(F)** TLS gene signature and TMB score in low grade gliomas (LGG, n=514), glioblastoma (GBM, n=151), uterine carcinosarcoma (UCS, n=57), kidney renal clear cell carcinoma (KIRC, n=368), kidney renal papillary cell carcinoma (KIRP, n=282), uterine corpus endometrial carcinoma (UCEC, 525), ovarian carcinoma (OV, n=207), rectum adenocarcinoma (READ, n=124), colon adenocarcinoma (COAD, n=364), lung adenocarcinoma (LUAD, n=507), lung squamous cell carcinoma (LUSC, n=467), skin cutaneous melanoma (SKCM, n=471) patients from the TCGA public database. Statistical significance was calculated by two-sided Mann-Whitney test. *p* values are indicated.

Supplementary Tables

Supplementary Table 1. **The main clinicopathological characteristics of 209 HGSOC patients from study cohort 1** (Study group 1 and 2) **and cohort 2** (Study group 3) **employed within survival analyses (Fig. 2G, H).**

Variable	Study group 1 (n=123)	Study group 2 (n=60)	Study group 3 (n=26)	Overall (n=209)
**Age**				
Mean Age	60	57	61	59
Range	37-79	33-81	46-75	33-81
**pTNM stage**				
Stage I	24 (19.5%)	1 (1.7%)	0 (0%)	25 (12%)
Stage II	10 (8.1%)	3 (5%)	0 (0%)	13 (6%)
Stage III	79 (64.2%)	53 (88.3%)	26 (100%)	158 (75.5%)
Stage IV	10 (8.2%)	3 (5%)	0 (0%)	13 (6.5%)
**Debulking**				
R0	63 (51.2%)	27 (45%)	22 (84.6%)	112 (53.5%)
R1	9 (7.3%)	7 (11.6%)	4 (15.4%)	20 (9.5%)
R2	51 (41.5%)	26 (44%)	0 (51.7%)	77 (37%)
**Vital status of patients**				
Alive	46 (36%)	27 (45%)	15 (57.7%)	88 (42%)
Death	77 (64%)	33 (55%)	11 (42.3%)	121 (58%)

Supplementary Table 2. **The main clinicopathological characteristics of 79 HGSOC patients from study cohort 2.**

Variable	Study Group 3 (n=26)	Study Group 4 (n=53)	Overall (n=79)
**Age**			
Mean Age	61	58	59
Range	46-75	24-73	24-75
**pTNM stage**			
Stage I	0 (0%)	0 (0%)	0 (0%)
Stage II	0 (0%)	0 (0%)	0 (0%)
Stage III	26 (100%)	53 (100%)	79 (100%)
Stage IV	0 (0%)	0 (0%)	0 (0%)
**Debulking**			
R0	22 (84.6%)	45 (51.2%)	67 (84%)
R1	4 (15.4%)	8 (7.3%)	12 (16%)
R2	0 (51.7%)	0 (41.5%)	0 (0%)
**Vital status of patients**			
Alive	15 (57.7%)	34 (36%)	49 (62%)
Death	11 (42.3%)	19 (64%)	30 (38%)

Abbreviations. HGSOC, high grade serous ovarian carcinoma

Supplementary Table 3. **Univariate Cox proportional hazard analyses in early (A) and late stage (B) HGSOC patients (Study cohort 1)**. Statistical significance was calculated by Univariate cox proportional hazard analyses. HR and *p* values are indicated.

A

		RFS			OS	
**Variable**		**HR (95%CI)**	***p* value**		**HR (95%CI)**	***p* value**
**TLS clusters**	CL 1	1		CL 1	1	
	CL 2	0.2 (0.08-0.53)	**0.001**	CL 2	0.53 (0.17-1.68)	0.279
	CL 3	0.85 (0.26-2.78)	0.783	CL 3	1.55 (0.41-5.85)	0.521
**Stage**	I	1		I	1	
	II	1.47 (0.63-3.45)	0.371	II	0.71 (0.24-2.07)	0.524
**Debulking**	R0	1		R0	1	
	R2	2.77 (0.64-12.05)	0.174	R2	2.89 (0.63-13.13)	0.171
**Age**		1.07 (1.02-1.12)	**0.003**		1.047 (1.01-1.13)	**0.015**

B

		RFS			OS	
**Variable**		**HR (95%CI)**	***p* value**		**HR (95%CI)**	***p* value**
**TLS clusters**	CL 1	1		CL 1	1	
	CL 2	0.55 (0.37-0.81)	**0.002**	CL 2	0.58 (0.37-0.90)	**0.014**
	CL 3	1.07 (0.66-1.73)	0.797	CL 3	1.07 (0.64-1.78)	0.803
**Stage**	III	1		III	1	
	IV	1.03 (0.54-1.96)	0.939	IV	0.9 (0.42-1.94)	0.785
**Debulking**	R0	1		R0	1	
	R1	1.65 (0.95-2.88)	0.076	R1	1.84 (1.02-3.34)	**0.043**
	R2	1.63 (1.12-2.38)	**0.01**	R2	1.49 (0.98-2.25)	0.062
**Age**		1.02 (1.00-1.03)	**0.03**		1.03 (1.01-1.05)	**0.004**

Abbreviations. TLS, tertiary lymphoid structures

Supplementary Table 4. **Multivariate Cox proportional hazard analyses in early (A) and late stage (B) HGSOC patients (Study cohort 1).** Statistical significance was calculated by Multivariate cox proportional hazard analyses. HR and *p* values are indicated.

A

		RFS			OS	
**Variable**		**HR (95%CI)**	***p* value**		**HR (95%CI)**	***p* value**
**TLS clusters**	CL 1			CL 1		
	CL 2	0.25 (0.08-0.77)	**0.015**	CL 2	0.8 (0.21-2.99)	0.736
	CL 3	0.63 (0.10-4.09)	0.627	CL 3	2.08 (0.22-19.84)	0.523
**Debulking**	R0			R0		
	R2	0.68 (0.13-3.54)	0.651	R2	1.59 (0.26-9.57)	0.614
**Age**		1.06 (1.00-1.12)	**0.042**		1.06 (1.00-1.13)	**0.056**

B

		RFS			OS	
**Variable**		**HR (95%CI)**	***p* value**		**HR (95%CI)**	***p* value**
**TLS clusters**	CL 1	1		CL 1	1	
	CL 2	0.58 (0.39-0.88)	**0.011**	CL 2	0.63 (0.45-1.16)	**0.042**
	CL 3	0.96 (0.51-1.79)	0.886	CL 3	1.58 (0.79-3.12)	0.193
**Debulking**	R0	1		R0	1	
	R1	1.49 (0.85-2.62)	0.166	R1	1.6 (0.87-2.92)	**0.128**
	R2	1.49 (1.01-2.19)	**0.041**	R2	1.38 (0.9-2.11)	0.135
**Age**		1.01 (1.00-1.03)	**0.153**		1.02 (1.00-1.04)	**0.058**

Abbreviations. TLS, tertiary lymphoid structures

Supplementary Table 5. **The main clinicopathological characteristics of 40 HGSOC patients involved in the study cohort 3 (validation cohort).**

Variable	Overall cohort (no=40)
**Age**	
Mean Age	61
Range	35-87
**pTNM stage**	
Stage I	3 (8%)
Stage II	4 (10%)
Stage III	26 (64%)
Stage IV	7 (18%)
**Vital status of patients**	
Non-relaps	27 (70%)
Relaps	13 (30%)

Supplementary Table 6. **The main clinicopathological characteristics of 31 stage III NSCLC patients involved in the study cohort 5.**

Variable	Overall cohort (no=31)
**Age**	
Mean Age	66
Range	42-79
**Histology**	
ADC	31 (100%)
SCC	0 (0%)
Others	0 (0%)
**pTNM stage**	
Stage I	0 (0%)
Stage II	0 (0%)
Stage III	31 (100%)
**Smoking status**	
Current	24 (77%)
Never Smoker	6 (23%)
**Vital status of patients**	
Alive	13 (58%)
Death	18 (42%)

Abbreviations: ***ADC***, adenocarcinoma; ***SCC***, squamous cell lung carcinoma.

Supplementary Table 7. **The main clinicopathological characteristics of 12 HGSOC patients involved in the study cohort 6.**

Variable	Overall cohort (no=12)
**Age**	
Mean Age	65
Range	58-78
**pTNM stage**	
Stage I	0 (0%)
Stage II	0 (0%)
Stage III	11 (92%)
Stage IV	1 (8%)
**Debulking**	
R0	7 (58%)
R1	2 (17%)
R2	3 (25%)
**Vital status of patients**	
Alive	12 (100%)
Death	0 (0%)

Supplementary Table 8. **The main clinicopathological characteristics of 7 stage III NSCLC patients involved in the study cohort 7.**

Variable	Overall cohort (no=7)
**Age**	
Mean Age	69
Range	48-71
**Histology**	
ADC	7 (100%)
SCC	0 (0%)
Others	0 (0%)
**pTNM stage**	
Stage I	0 (0%)
Stage II	0 (0%)
Stage III	7 (100%)
**Smoking status**	
Current	3 (71%)
Never Smoker	2 (29%)
**Vital status of patients**	
Alive	7 (100%)
Death	0 (0%)

Abbreviations: ***ADC***, adenocarcinoma; ***SCC***, squamous cell lung carcinoma.

Supplementary Table 9. **Antibodies and detection systems used for immunohistochemistry and immunofluorescence microscopy.**

Parameter	Source	Producer	Clone	Detection system	Revelation	Dilution	Incubation time [min]
**Immunohistochemistry**							
**CD3***	rabbit	DAKO	NA	Impress HRP anti-mouse IgG (Peroxidase) Polymer Detection kit	DAB+ substrate Chromogen system	1:80	90
**CD4**	mouse	Leica Biosystems	4B12	Impress EXCEL Amplified anti-rabbit IgG kit	DAB+ substrate Chromogen system	1:100	60
**CD4**	rabbit	Abcam	EPR19514	Impress HRP anti-rabbit IgG (Peroxidase) Polymer Detection kit	AEC+ substrate Chromogen system	1:150	60
**CD8**	rabbit	Abcam	CAL38	Impress EXCEL Amplified anti-rabbit IgG kit	DAB+ substrate Chromogen system	1:250	60
**CD8**	rabbit	Spring Bioscience	SP16	EnVision™+/HRP, Rabbit	DAB+ substrate Chromogen system	1:80	30
**CD20**	rabbit	Abcam	SP32	Impress HRP anti-rabbit IgG (Peroxidase) Polymer Detection kit	AEC+ substrate Chromogen system	1:100	60
**CD20***	mouse	Dako	L26	ImmPRESS-AP anti-mouse IgG (alkaline phosphatase) Polymer Detection Kit	ImmPACT Vector red Alkaline Phosphatase substrate kit	1:250	60
**CD20**	rabbit	Abcam	SP32	Impress HRP anti-rabbit IgG (Peroxidase) Polymer Detection kit	DAB+ substrate Chromogen system	1:100	60
**CD21**	rabbit	Abcam	EP3093	Impress HRP anti-rabbit IgG (Peroxidase) Polymer Detection kit	AEC+ substrate Chromogen system	1:150	60
**CD21/CR2**	mouse	Cell signaling	2G9	Impress HRP anti-mouse IgG (Peroxidase) Polymer Detection kit	AEC+ substrate Chromogen system	1:50	60
**CD23**	rabbit	Abcam	SP23	ImmPRESS-AP anti-rabbit IgG (alkaline phosphatase) Polymer Detection Kit	AEC+ substrate Chromogen system	1:100	60
**CD57**	mouse	Abcam	NK/804	Impress HRP anti-mouse IgG (Peroxidase) Polymer Detection kit	AEC substrate system	1:50	120
**CTLA-4**	mouse	Santa cruz biotechnology	F-8	Impress HRP anti-mouse IgG (Peroxidase) Polymer Detection kit	DAB+ substrate Chromogen system	1:200	110
**DC-LAMP***	rat	Dendritics	1010E1.01	donkey anti-rat IgG-biot (Jackson ImmunoResearch)	DAB+ substrate Chromogen system	1:80	60
**FoxP3**	mouse	Abcam	236A/E7	ImmPRESS-AP anti-mouse IgG (alkaline phosphatase) Polymer Detection Kit	Vector blue	1:80	90
**LAG-3**	mouse	Abcam	17B4	Impress HRP anti-mouse IgG (Peroxidase) Polymer Detection kit	DAB+ substrate Chromogen system	1:150	90
**NKp46**	mouse	RD systems	195314	Impress HRP anti-mouse IgG (Peroxidase) Polymer Detection kit	DAB+ substrate Chromogen system	1:100	90
**PD1**	mouse	Abcam	NAT105	Impress HRP anti-mouse IgG (Peroxidase) Polymer Detection kit	DAB+ substrate Chromogen system	1:50	60
**PanCK**	mouse	Abcam	AE1/AE3	Impress HRP anti-mouse IgG (Peroxidase) Polymer Detection kit	AEC substrate system	1:150	60

**TCF1/TCF7**	rabbit	Cell signaling	C63D9	Impress HRP anti-rabbit IgG (Peroxidase) Polymer Detection kit	AEC substrate systém	1:50	120
**TIM3**	rabbit	Cell signaling	D5D5R	Jackson Immuno Research, Donkey anti Rabbit IgG	AEC substrate system	1:50	120

***CD20/DC-LAMP and CD3/FoxP3 were stained in double staining protokol**

Immunofluorescence

**CD8**	rabbit	Abcam	SP16	poly-HRP-conjugated sec.Ab goat anti-rabbit	Alexa Fluor™ 594 Tyramide reagent	1:60	90
**CD20**	mouse	Dako	L26	goat anti-mouse IgG (H+L cross absorbed secondary antobody A750		1:300	30
**CD21/CR2**	mouse	Cell signaling	2G9	poly-HRP-conjugated sec.Ab goat anti-mouse	Alexa Fluor™ 555 Tyramide reagent	1:150	60
**CD23**	rabbit	Abcam	SP23	poly-HRP-conjugated sec. Ab goat anti-rabbit	Alexa Fluor™ 488 Tyramide reagent	1:500	60
**DC-LAMP**	rat	Dendritics	1010E1.01	Immpress -HRP anti-rat IgG polymer	Alexa Fluor™ 546 Tyramide reagent	1:350	overnight
**GZMB**	rabbit	Abcam	EPR8260	poly-HRP-conjugated sec.Ab goat anti-rabbit	Alexa Fluor™ 488 Tyramide reagent	1:250	60

Ultivue

**CD8**	na	Ultivue	C8/144B	FITC	Ultivue (manufacturer´s instructions)
**PD1**	na	Ultivue	CAL 20	TRIC	Ultivue (manufacturer´s instructions)
**PD-L1**	na	Ultivue	73-10	Cy5	Ultivue (manufacturer´s instructions)
**CD68**	na	Ultivue	KP-1	Cy7	Ultivue (manufacturer´s instructions)
**GZMB**	na	Ultivue	EPR8260	FITC	Ultivue (manufacturer´s instructions)
**CD4**	na	Ultivue	SP35	TRIC	Ultivue (manufacturer´s instructions)
**FoxP3**	na	Ultivue	236A/E7	Cy5	Ultivue (manufacturer´s instructions)
**CD20**	na	Ultivue	L26	Cy7	Ultivue (manufacturer´s instructions)

***Supplementary Table 10*. Antibodies used for flow cytometry.**

Parameter	Source	Producer	Clone	Fluorochrome	Dilution
**CCR7**	mouse	BioLegend	G043H7	PerCP-Cy 5.5	6:100
**CD3**	mouse	Exbio	MEM-57	A700	5:100
**CD4**	mouse	Thermo Fisher Scientific	RPA-T4	PE-Cy 7	4:100
**CD8**	mouse	BD Biosciences	RPA-T8	HV 500	5:100
**CD14**	mouse	Beckman Coulter	RM052	ECD	6:100
**CD19**	mouse	BD Biosciences	SJ25C1	PE-Cy 7	4:100
**CD20**	mouse	Exbio	LT20	FITC	6:100
**CD45**	mouse	Exbio	MEM-28	PerCP	4:100
**CD45**	mouse	Thermo Fisher Scientific	HI30	PE-Texas Red	6:100
**CD45 RO**	mouse	Exbio	UCHL1	APC	6:100
**CD56**	mouse	BioLegend	HCD-56	BV 421	5:100
**EPCAM**	mouse	BioLegend	9C4	PE	3:100
**PD1**	mouse	BioLegend	EH12.2H7	FITC	6:100
**TIM3**	mouse	BioLegend	F38-2E2	PE	3:100

References:

1. Angelova M, Charoentong P, Hackl H, Fischer ML, Snajder R, Krogsdam AM, *et al.* Characterization of the immunophenotypes and antigenomes of colorectal cancers reveals distinct tumor escape mechanisms and novel targets for immunotherapy. Genome Biol **2015**;16:64 doi 10.1186/s13059-015-0620-6.
2. King HW, Orban N, Riches JC, Clear AJ, Warnes G, Teichmann SA, *et al.* Single-cell analysis of human B cell maturation predicts how antibody class switching shapes selection dynamics. Sci Immunol **2021**;6(56) doi 10.1126/sciimmunol.abe6291.
3. Keane TM, Goodstadt L, Danecek P, White MA, Wong K, Yalcin B, *et al.* Mouse genomic variation and its effect on phenotypes and gene regulation. Nature **2011**;477(7364):289-94 doi 10.1038/nature10413.
4. Dobin A, Davis CA, Schlesinger F, Drenkow J, Zaleski C, Jha S, *et al.* STAR: ultrafast universal RNA-seq aligner. Bioinformatics **2013**;29(1):15-21 doi 10.1093/bioinformatics/bts635.
5. Kim S, Scheffler K, Halpern AL, Bekritsky MA, Noh E, Kallberg M, *et al.* Strelka2: fast and accurate calling of germline and somatic variants. Nat Methods **2018**;15(8):591-4 doi 10.1038/s41592-018-0051-x.
