# Supplementary material for: Tertiary lymphoid structures and B cells determine clinically relevant T cell phenotypes in ovarian cancer
Source: Nat Commun. 2024 Mar 21;15:2528. doi: 10.1038/s41467-024-46873-w (PMC10957872; doi:10.1038/s41467-024-46873-w)
Supplement: Supplementary file 3 — Description of Additional Supplementary Files [file 41467_2024_46873_MOESM3_ESM.pdf]

Description of Additional Supplementary Files

Supplementary Data 1: Genes differentially represented in tumor HGSOC samples (Study group 1) containing eTLSs only (Cluster 2 [CL2]) (A) and eTLSs plus mTLSs (Cluster 3 [CL3]) (B) vs containing no TLSs within TME (Cluster 1 [CL1]).
